# Randomized controlled trial on the effect of 1‐hour infusion of vincristine versus push injection on neuropathy in children with cancer (final analysis)

**DOI:** 10.1002/cam4.6550

**Published:** 2023-09-21

**Authors:** Aniek Uittenboogaard, Marleen H. van den Berg, Floor C. H. Abbink, Jos W. R. Twisk, Inge M. van der Sluis, Cor van den Bos, Marry M. van den Heuvel‐Eibrink, Heidi Segers, Christophe Chantrain, Jutte van der Werff ten Bosch, Leen Willems, Gertjan J. L. Kaspers, Mirjam Esther van de Velde

**Affiliations:** ^1^ Pediatric oncology Emma Children's Hospital Amsterdam UMC Vrije Universiteit Amsterdam Amsterdam the Netherlands; ^2^ Princess Máxima Center for Pediatric Oncology Utrecht the Netherlands; ^3^ Department of Epidemiology and Biostatistics Amsterdam UMC Vrije Universiteit Amsterdam Amsterdam the Netherlands; ^4^ Department of Pediatric Oncology Erasmus Medical Center Rotterdam/Sophia Children's Hospital Rotterdam the Netherlands; ^5^ Department of Pediatric Hemato‐Oncology University Hospitals Leuven and Catholic University Leuven Leuven Belgium; ^6^ Department of Pediatrics Clinique du MontLégia The Centre Hospitalier Chrétien Liège Belgium; ^7^ Department of Pediatric Onco‐Hematology Universitair Ziekenhuis Brussel Brussels Belgium; ^8^ Department of Paediatric Haematology‐Oncology and Stem Cell Transplantation Ghent University Hospital Ghent Belgium

**Keywords:** cancer, children, toxicity, vincristine

## Abstract

**Introduction:**

Vincristine is an integral component of treatment for children with cancer. Its main dose‐limiting side effect is vincristine‐induced peripheral neuropathy (VIPN). The VINCA trial was a randomized controlled trial that explored the effect of 1‐hour infusion compared with push injection of vincristine on the development of VIPN in children with cancer. The short‐term outcomes (median follow‐up 9 months) showed that there was no difference in VIPN between the randomization groups. However, 1‐hour infusion was less toxic in children who also received azoles. We now report the results of the final analyses (median follow‐up 20 months), which includes treatment outcome as a secondary objective (follow‐up 3 years).

**Methods:**

VIPN was measured 1–7 times per participant using the Common Terminology Criteria for Adverse Events (CTCAE) and the pediatric‐modified total neuropathy score. Poisson mixed model and logistic generalized estimating equation analysis for repeated measures were performed.

**Results:**

Forty‐five participants per randomization group were included. There was no significant effect of 1‐hour infusion compared with push injection on VIPN. In participants receiving concurrent azoles, the total CTCAE score was significantly lower in the one‐hour group (rate ratio 0.52, 95% confidence interval 0.33–0.80, *p* = 0.003). Four patients in the one‐hour group and one patient in the push group relapsed. Two patients in the one‐hour group died.

**Conclusion:**

1‐hour infusion of vincristine is not protective against VIPN. However, in patients receiving concurrent azoles, 1‐hour infusion may be less toxic. The difference in treatment outcome is most likely the result of differences in risk profile.

## Abstract 2

Vincristine is an integral component of treatment for children with cancer. Its main dose‐limiting side effect is vincristine‐induced peripheral neuropathy (VIPN). The VINCA trial was a randomized controlled trial that explored the effect of one‐hour infusion compared with push injection of vincristine on the development of VIPN in children with cancer. We found that in patients receiving concurrent azoles, one‐hour infusion may be less toxic.

## 1 INTRODUCTION

Vincristine is a chemotherapeutic agent that is frequently used in childhood cancer treatment protocols, such as those for acute lymphoblastic leukemia (ALL), lymphoma, and nephroblastoma.^1^, ^2^, ^3^ Its main side effect is vincristine‐induced peripheral neuropathy (VIPN), which often manifests as a symmetric sensory‐motor neuropathy with a stocking‐and‐glove distribution.^1^, ^2^, ^3^ Symptoms of VIPN typically develop 1 week or more after starting vincristine treatment and may persevere after treatment. Indeed, a study in ALL survivors described that 16% suffered from VIPN long‐term, which significantly impacted the quality of life.^4^

Severe VIPN is estimated to affect up to 30% of patients, but it remains a challenge to predict which patients are at risk.^5^ Some risk factors have been identified; for instance, White patients appear to be more affected than Black patients.^6^, ^7^, ^8^, ^9^ Age is another risk factor: children over 10 years old appear to have more VIPN than younger patients.^2^, ^7^, ^10^ Moreover, concurrent treatment with azoles may increase the risk of VIPN.^1^, ^2^ Azoles inhibit CYP3A4, which may affect hepatic clearance of vincristine, resulting in prolonged exposure and thus higher toxicity.^1^

The treatment options for VIPN are limited to pain medication and reducing vincristine dosage.^1^, ^2^ Since the latter can result in lower antitumor activity, alternative strategies are warranted. One study found that a higher maximum plasma concentration (C_max_) was associated with more autonomic neuropathy.^11^ Increasing administration duration avoids a high C_max._ ^12^ Indeed, two studies reported that if given as a continuous infusion over 4–5 days, vincristine dosages of 3.5–4.0 mg/m^2^ were well tolerated without an increase in VIPN incidence.^13^, ^14^ However, multi‐day infusions are costly and cumbersome. 1‐hour infusion may be more feasible, avoid a high C_max_ ^12^ and thus have a protective effect against toxicity.

The VINCA trial is a randomized controlled trial (RCT) on the effect of 1‐hour infusion compared with push injection on VIPN in children with cancer. In the first report of the VINCA trial (median follow‐up 9 months), we found no effect of administration duration of vincristine on VIPN. However, in participants receiving concurrent azoles, 1‐hour infusion resulted in less VIPN compared with push injection. In this final follow‐up study (median follow‐up 20 months), we assess the long‐term effect of vincristine administration duration on the development of VIPN. As a secondary objective, we assessed the effect on treatment outcome (follow‐up 3 years).

## 2 METHODS

### 2.1 Patients

The design and methods of the VINCA trial were published previously.^15^ In short, the VINCA trial was an international multicenter open‐label RCT in which participants were randomized between push and one‐hour administration of vincristine. Stratified block randomization was applied for age (2–10 years or 11–18 years), sex, and country. Newly diagnosed participants aged between 2 and 18 years without pre‐existing peripheral neuropathy were included in this study. Patients with the following diagnoses and treatment plans were eligible for inclusion: ALL (DCOG ALL‐11 treatment protocol,^16^ EsPhALL protocol,^17^ or EORTC‐58081‐CLG guideline^18^), Hodgkin's lymphoma (EuroNet‐PHL‐C1 protocol^19^ or C2 protocol^20^), nephroblastoma (SIOP Wilms 2001 protocol^21^), rhabdomyosarcoma (EpSSG RMS 2005 protocol^22^), low‐grade glioma (SIOP‐LGG 2004 protocol^23^), and medulloblastoma (ACNS0331^24^ or ACNS0332 protocol^25^). The Institutional Review Board (IRB) of Amsterdam UMC, location VUmc (IRB number: 2014–268, EUDRACT number: 2014–001561‐27) approved the study protocol. The study was conducted in accordance with the Declaration of Helsinki.

### 2.2 Assessment of VIPN

VIPN was assessed using the CTCAE, version 4.03^26^ and the pediatric‐modified total neuropathy score (ped‐mTNS).^27^, ^28^ The following CTCAE items were included: constipation (range 0–5), peripheral sensory neuropathy (range 0–5), peripheral motor neuropathy (range 0–5), and neuralgia (range 0–3). If participants scored ≥2 on any of these items, they were considered to have VIPN. A score of ≥3 on any of these items was defined as severe VIPN. Furthermore, the four items were summed in a total CTCAE score (max 18). The ped‐mTNS can be used in patients aged 5–18 years.^27^ Participants could score maximum 32 points and a score of ≥5 was defined as VIPN.^27^ VIPN was assessed at pre‐defined time points during treatment, including a measurement 6 months after ending vincristine treatment.^15^

### 2.3 Sample size calculation

The sample size calculation was based on total CTCAE scores.^15^ A difference in CTCAE score of at least 1.0 was considered to be clinically relevant. This resulted in a target sample size of at least 70 patients (35 patients per randomization group, *α* = 5%, *β* = 90%). With an expected drop‐out rate of 25%, we aimed to include 88 participants in total (44 per randomization group).

### 2.4 Statistical analysis

The statistical analysis was performed in R, version 4.0.3 (Rstudio Inc.).^29^ Participant characteristics and the overall incidence of VIPN per randomization group were described using frequency distributions, means with standard deviations (SD), and medians with interquartile ranges (IQR). We performed an intention‐to‐treat analysis. Furthermore, a per protocol analysis was performed in which participants were excluded who received <90% of the vincristine administrations differently than their allocated infusion time. For the effect of randomization group on the development of VIPN over time, with total CTCAE and ped‐mTNS scores, Poisson mixed model analysis for repeated measures was performed using the ‘lme4’ package.^30^ Randomization group and baseline total CTCAE or ped‐mTNS scores were included as fixed effects. Rate ratios were used to describe the effect of randomization group on total CTCAE and ped‐mTNS score, in which the rate ratio corresponds to an increase of one point on the CTCAE or ped‐mTNS. For dichotomized VIPN scores, generalized estimating equation (GEE) analysis for repeated measures was performed using the ‘gee’ package, resulting in odds ratios (OR).^31^ We reported crude results and results adjusted for the following covariates: time period of VIPN assessment, age, sex, diagnosis, and racial background. Confounding and effect modifying effects of these covariates were assessed. Concurrent azoles was defined as follows: participants who received azoles in the week preceding or following vincristine administration in ≥50% of vincristine administrations between two VIPN study measurements. Time period of VIPN assessment was defined as follows: we combined all VIPN measurements during vincristine administration and all VIPN measurements ≥6 months after last vincristine administration. A two‐sided *p*‐value of ≤0.05 was considered statistically significant. The reproducible code of the statistical analysis is available in Data S1.

## 3 RESULTS

### 3.1 Participant characteristics

**FIGURE 1:**
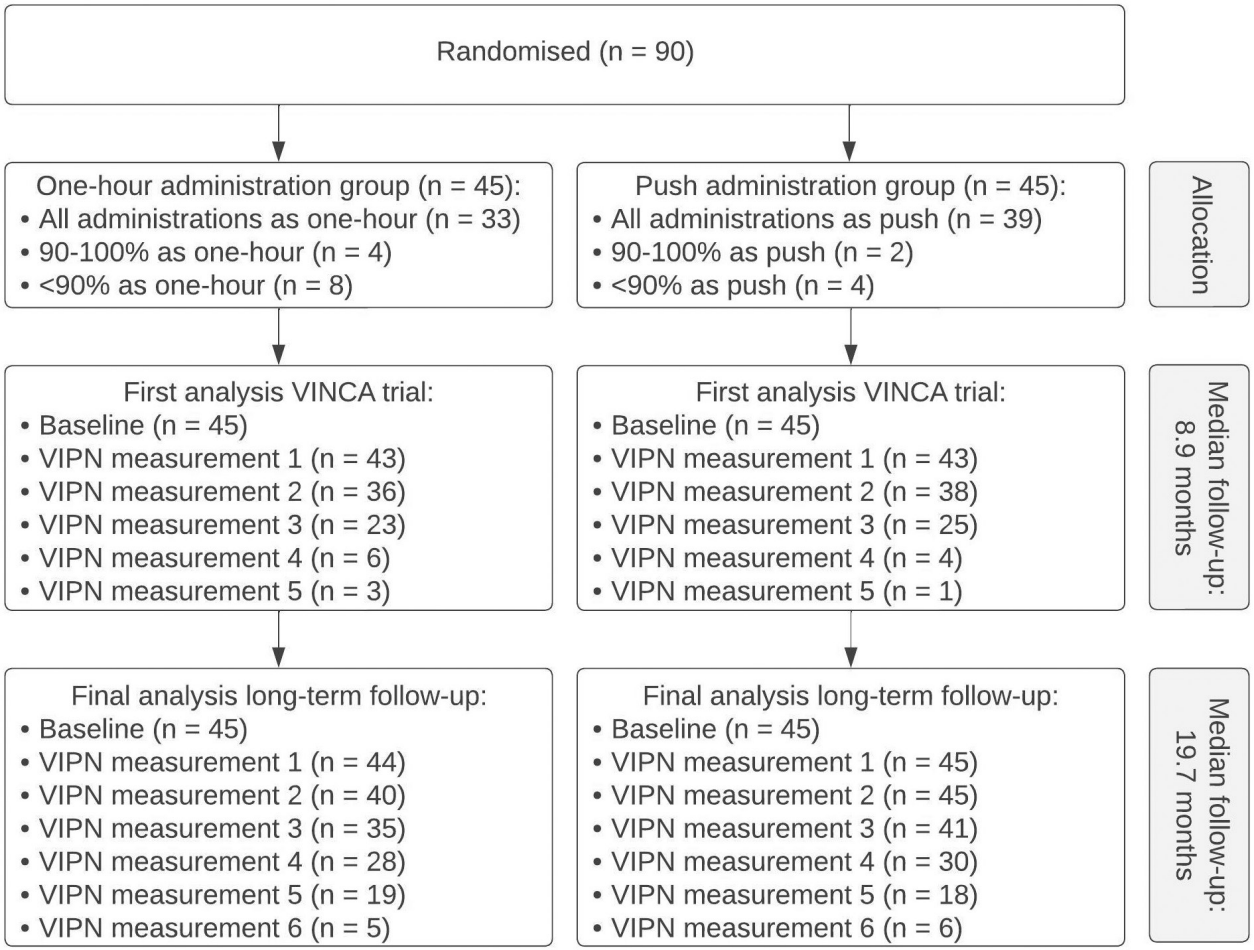
Flow diagram of the participants, including the number of measurements in the first analysis of the VINCA trial and the extended follow‐up study. The full flow diagram of the screening, randomization, and follow‐up of the VINCA study can be found in the first analysis of the VINCA trial.^15^ VIPN, vincristine‐induced peripheral neuropathy.

Figure 1 shows the enrollment and follow‐up of the participants in this extended follow‐up study. Ninety patients participated in the VINCA trial, of which 45 were randomized to one‐hour administration of vincristine. The majority of participants were White, diagnosed with ALL, and between 2 and 10 years at the start of the study (Table 1). Eleven children in the push group and 12 children in the one‐hour group were younger than 5 years at the start of the study (Table S1). VIPN measurements were performed maximum six times after the first baseline measurement (Figure 1). The mean follow‐up duration was 21.0 months (SD 9.6 months) for the push group and 18.3 months (SD 10.1) for the one‐hour group (*p* = 0.19). During follow‐up, participants received a mean body surface area (BSA) normalized cumulative vincristine dosage of 22.6 (SD 15.8) and 19.4 (SD 13.6) mg/m^2^ for the push and one‐hour group, respectively (*p* = 0.29). Per randomization group, seven participants received azoles during vincristine administration. No patients in the push group required vincristine dose reduction or omission, whereas this was needed for two patients in the one‐hour group (*p* = 0.49).

**TABLE 1:** Characteristics of participants in the randomization groups (push‐ and one‐hour administration of vincristine).

	Push administration group (*n* = 45)	One‐hour administration group (*n* = 45)
Male, *n* (%)	24 (53.3)	26 (57.8)
Disease, *n* (%)		
ALL	29 (64.4)	29 (64.4)
Hodgkin lymphoma	11 (24.4)	7 (15.6)
Medulloblastoma	1 (2.2)	1 (2.2)
Low‐grade glioma	2 (4.4)	0 (0)
Wilms tumor	2 (4.4)	6 (13.3)
Rhabdomyosarcoma	0 (0)	2 (4.4)
Age at start study, *n* (%)		
2–10 years	27 (60)	27 (60)
11–18 years	18 (40)	18 (40)
Racial background, *n* (%)		
White	37 (82.2)	36 (80)
Non‐White	8 (17.8)	9 (20)
Follow‐up duration in months (mean [SD])	21.0 (9.6)	18.3 (10.1)
BSA normalized cumulative vincristine dosage during follow‐up in mg/m^2^ (mean [SD])	22.6 (15.8)	19.4 (13.6)
Vincristine dose reduction or omission, *n* (%)	0 (0)	2 (4.4)

Abbreviations: ALL, acute lymphoblastic leukemia; BSA, body surface area; SD, standard deviation.

### 3.2 CTCAE

**FIGURE 2:**
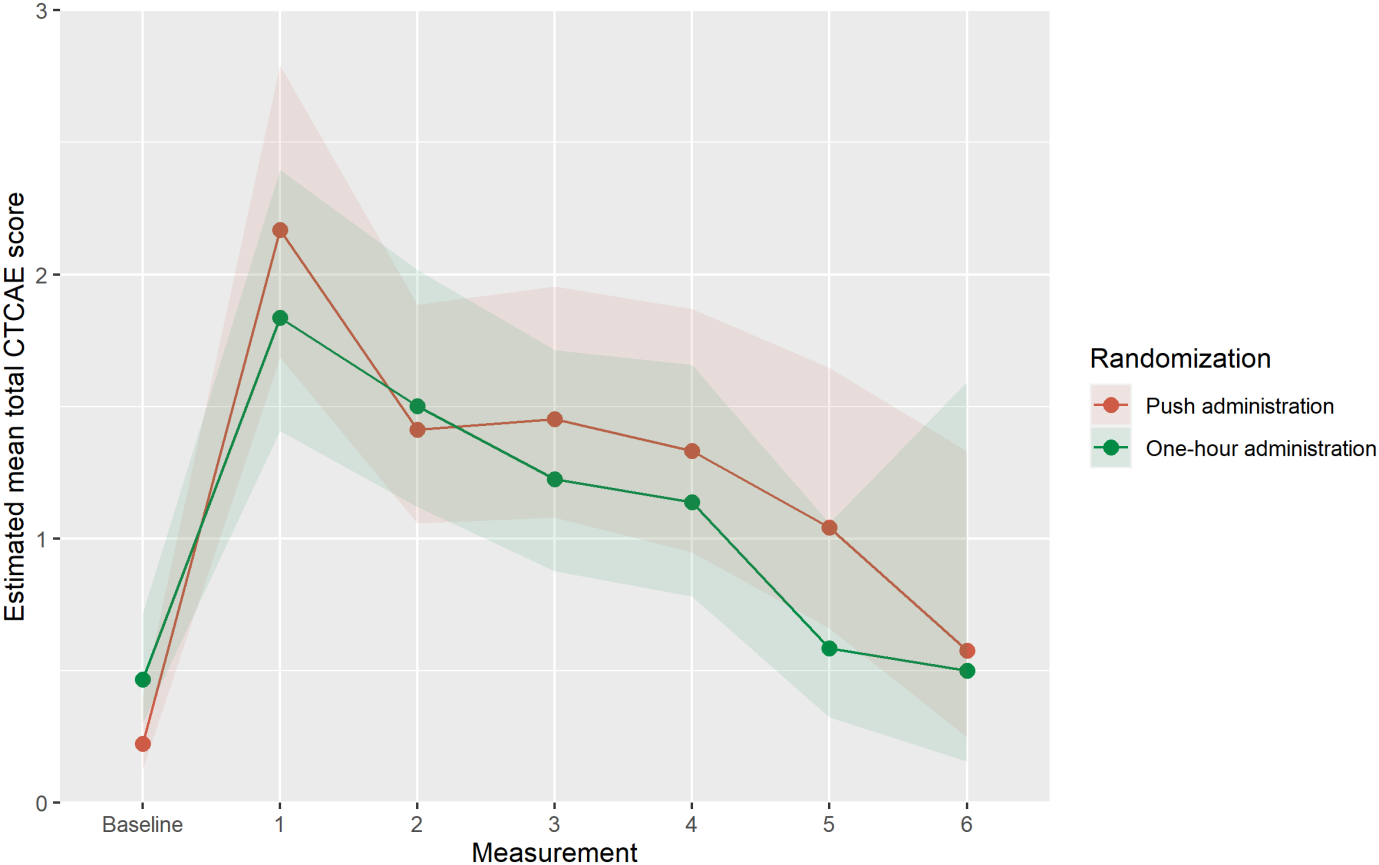
Estimated total CTCAE score over time in the randomization groups including 95% confidence interval (corrected for baseline). Median days and IQR per measurement moment are as follows: measurement 1: push = 29 (27; 42) and one‐hour = 28 (25; 47), measurement 2: push = 163 (129; 218) and one‐hour = 167 (99.5; 208), measurement 3: push = 403 (276; 518) and one‐hour = 387 (228; 466), measurement 4: push = 638 (529; 715) and one‐hour = 644 (410.5; 666), measurement 5: push = 915 (679.3; 957) and one‐hour = 884 (500; 953), and measurement 6: push = 868 (625.3; 895) and one‐hour = 469 (426.5–827). For the number of participants per measurement moment: see Figure 1.

According to the CTCAE, 57.8 and 55.6% of the push and one‐hour group developed VIPN, of which 15.6 and 13.3% developed severe VIPN, respectively (Grade 3 or higher) (Table 2). The median number of follow‐up measurements was four in both the push and one‐hour group (IQR 3–5). The development of VIPN over time according to the CTCAE was not significantly different between the randomization groups (Table 3). Figure 2 illustrates the estimated mean total CTCAE scores over time for both randomization groups. The findings were similar in the analysis adjusted for age, sex, BSA normalized cumulative vincristine dosage, diagnosis, and racial background (Table S2).

**TABLE 2:** Incidence of vincristine‐induced peripheral neuropathy of participants in the randomization groups (push‐ and one‐hour administration of vincristine).

	Push group (*n* = 45), *n* (%)	One‐hour group (*n* = 45), *n* (%)	*p*‐value
VIPN based on CTCAE	26 (57.8)	25 (55.6)	0.83
Severe VIPN based on CTCAE	7 (15.6)	6 (13.3)	0.76
VIPN based on ped‐mTNS*	24 (72.7)	24 (64.9)	0.48
CTCAE score (median [IQR])	1.00 (0.00; 2.00)	1.00 (0.00; 2.00)	0.96
Ped‐mTNS score (median [IQR])	2.00 (0.00; 6.00)	2.00 (0.00; 5.00)	0.68

VIPN outcomes without concurrent azole antifungals	Push group (*n* = 38)	One‐hour group (*n* = 38)	*p*‐value
CTCAE score (median [IQR])	1.00 (0.00; 3.00)	1.00 (0.00; 2.00)	0.74
Ped‐mTNS score (median [IQR])**	2.00 (0.00; 6.00)	3.00 (1.00; 7.00)	0.93

VIPN outcomes with concurrent azole antifungals	Push group (*n* = 7)	One‐hour group (*n* = 7)	*p*‐value
CTCAE score (median [IQR])	1.00 (0.00; 2.00)	1.50 (0.25; 3.00)	0.99
Ped‐mTNS score (median [IQR])***	2.00 (0.50; 10.50)	5.00 (0.00; 9.00)	0.45

* Total group consisted of 70 participants (push group: 33, one‐hour group: 37)

** Total group consisted of 59 participants (push group: 28, one‐hour group: 31)

*** Total group consisted of 11 participants (push group: 5, one‐hour group: 6).

Abbreviations: CTCAE, common terminology of adverse events; IQR, interquartile range; ped‐mTNS, pediatric‐modified Total Neuropathy Score; VIPN, vincristine‐induced peripheral neuropathy.

**TABLE 3:** The effect of one‐hour administration in comparison with push administration of vincristine on the development of vincristine‐induced peripheral neuropathy over time.

	Total group (*n* = 89)		Subgroup of participants without concurrent azole antifungals (*n* = 75)		Subgroup of participants with concurrent azole antifungals (*n* = 14)	
Continuous outcomes	Rate ratio (95% CI)	*p*‐value	Rate ratio (95% CI)	*p*‐value	Rate ratio (95% CI)	*p*‐value
Total CTCAE	0.92 (0.67–1.26)	0.59	1.03 (0.72–1.47)	0.88	0.51 (0.33–0.80)	0.003
Total ped‐mTNS*	0.98 (0.63–1.53)	0.93	1.03 (0.61–1.75)	0.91	1.00 (0.60–1.70)	0.97

Dichotomized outcomes	OR (95% CI)	*p*‐value	OR (95% CI)	*p*‐value	OR (95% CI)	*p*‐value
CTCAE	0.74 (0.41–1.33)	0.32	0.87 (0.45–1.67)	0.67	0.34 (0.08–1.49)	0.15
Ped‐mTNS*	1.02 (0.52–2.01)	0.95	0.99 (0.45–2.13)	0.97	1.10 (0.35–3.49)	0.87
Severe VIPN according to CTCAE	0.59 (0.19–1.84)	0.36	1.03 (0.17–6.35)	0.97	0.26 (0.06–1.20)	0.09

*Note*: Reference group is push administration. CTCAE: 350 observations in 89 participants and ped‐mTNS: 221 observations in 60 participants. One participant could not be included in these analyses since only a baseline measurement was available.

* Total group consisted of 60 participants (without concurrent azole antifungals: 49, with concurrent azole antifungals: 11).

Abbreviations: CI, confidence interval; CTCAE, common terminology criteria for adverse events; OR, odds ratio; ped‐mTNS, pediatric modified total neuropathy score; VIPN, vincristine‐induced peripheral neuropathy.

**FIGURE 3:**
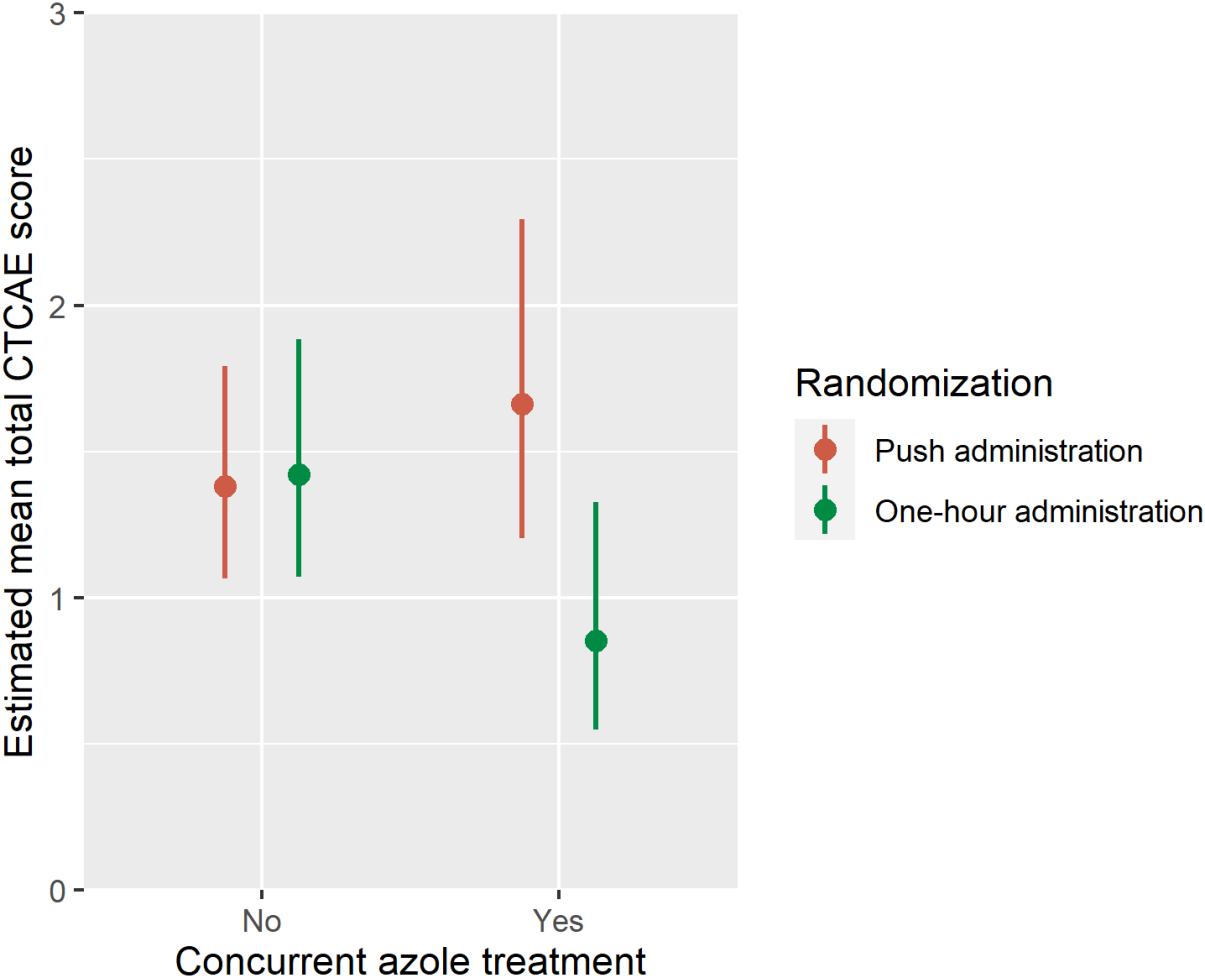
Estimated total CTCAE score over time in the randomization groups per concurrent azoles, including 95% confidence interval. Number of participants analyzed per group are as follows: push administration: *n* = 7 with concurrent azoles and *n* = 38 without concurrent azoles, one‐hour administration: *n* = 7 with concurrent azoles and *n* = 38 without concurrent azoles.

Age, sex, diagnosis, and racial background were not identified as modifiers in the relationship between VIPN according to CTCAE and randomization group. However, concurrent azoles was identified as a modifier in this relationship (*p* = 0.006). As an exploratory subgroup analysis, results are therefore reported separately for participants with and without concurrent azoles. In participants receiving concurrent azoles, the total CTCAE score was significantly lower in participants who received vincristine as a one‐hour administration compared with a push administration (rate ratio 0.52, 95% CI 0.33–0.80, *p* = 0.003) (Table 3 and Figure 3). The results remained similar in the adjusted analysis (rate ratio 0.40, 95% CI 0.24–0.66, *p* = 0.0003) (Table S2). Moreover, in this group, one‐hour administration of vincristine resulted in a borderline significantly lower risk of severe VIPN in comparison to push administration in the both the crude and adjusted analysis (OR 0.26, 95% CI 0.06–1.20, *p* = 0.09 and OR 0.17, 95% CI 0.02–1.27, *p* = 0.08) (Table 3 and Table S2).

No significant difference was found between the randomization groups all VIPN measurements during vincristine treatment and ≥6 months after last vincristine administration were compared (Figure S1).

The results were similar in the per protocol analyses (data not shown).

### 3.3 Ped‐mTNS

In the 70 participants of whom ped‐mTNS data were available (≥5 years old and 35 in each randomization group), respectively, 72.7 and 64.9% in the push and one‐hour groups developed VIPN. The median number of follow‐up measurements was four in the push group (IQR 2.25–4.75) and three in the one‐hour group (IQR 1.5–5). The development of VIPN over time according to the ped‐mTNS was not significantly different between the randomization groups, both in the crude and adjusted analyses (Table 3 and Table S2). This was the case for both total and dichotomized ped‐mTNS scores (Table 3 and Table S2).

Age, sex, concurrent azoles, diagnosis, and racial background were not identified as modifiers in the relationship between VIPN according to ped‐mTNS and randomization group.

The results were similar in the per protocol analyses (data not shown).

### 3.4 Treatment efficacy

At 3‐year follow‐up, one participant in the push group and six participants in the one‐hour group had an adverse treatment outcome (Table 4). Four patients in the one‐hour group and one patient in the push group relapsed. Two patients in the one‐hour group died during treatment: one due to disease progression and one due to treatment‐related toxicity in complete remission (hepatotoxicity, pulmonary and cerebral aspergillosis, severe neurotoxicity, and hemorrhagic shock). All participants with an adverse treatment outcome had a high to very high risk disease profile (Table 3 and Table S3).

**TABLE 4:** Characteristics of participants with an adverse treatment outcome.

Participant number	Randomization	Gender	Age at inclusion in years	Disease	BSA normalized cumulative vincristine dosage in mg/m^2^	First treatment outcome	Days since inclusion	Last treatment outcome	Days since inclusion
1	Push	Female	17	ALL HR	9.20	Relapse	Unknown	Death	254
2	One‐hour	Female	2	Nephroblastoma*	4.37	Death (disease progression)	50	N.A	N.A.
3	One‐hour	Male	12	ALL VHR	4.44	Death (treatment‐related toxicity)	132	N.A	N.A
4	One‐hour	Male	10	RMS HR	14.87	Relapse	383	Death	1105
5	One‐hour	Female	7	RMS HR	17.59	Relapse	222	Death	330
6	One‐hour	Female	6	Medulloblastoma HR	20.02	Relapse	797	Death	892
7	One‐hour	Male	2	ALL VHR	20.93	Relapse	1275	EFS	1679

Abbreviations: EFS, event‐free survival; HR, high risk; N.A, not applicable; RMS, rhabdomyosarcoma; VHR, very high risk.

* After initial diagnosis with a Wilms tumor, the participant received a different diagnosis during treatment (malignant rhaboid tumor of the kidney with pulmonary and lymph node metastases) and was subsequently excluded from the study. All vincristine administrations were given as 1‐hour infusion.

## 4 DISCUSSION

In this final analysis study, we assessed the long‐term effect of one‐hour administration of vincristine compared with push administration on the development of VIPN. We found that 1‐hour infusion of vincristine did not protect against VIPN compared with push injection, both during and 6 months after vincristine treatment. In an exploratory subgroup analysis, we found that one‐hour administration resulted was less toxic in children receiving concurrent azoles. More patients relapsed in the one‐hour group than in the push group, but this was most likely due to differences in disease risk profile.

Although in previous studies multi‐day infusions with dosages up to 3.5–4.0 mg/m^2^ could be given without additional toxicity,^13^, ^14^ 1‐hour infusion of vincristine might not be long enough to limit VIPN. This finding may be explained by the pharmacokinetics (PK), and specifically the distribution phase, of vincristine. Vincristine binds to β‐tubulins, causing cell arrest in the metaphase. In cancer cells, this results in the desired cytostatic effect, whereas in neurons, this can result in undesired VIPN.^32^, ^33^ However, tubulins are abundantly present in platelets and other blood cells as well.^34^, ^35^, ^36^ This has led to the hypothesis, confirmed by two population PK models, that upon entering the blood stream vincristine may bind rapidly to the tubulins in platelets and other blood cells.^37^, ^38^ This explains the rapid and extensive distribution phase of vincristine.^37^, ^38^ This β‐tubulin binding capacity appears saturable: if the tubulin compartment in the blood is saturated, the remaining overshoot of vincristine may exert the toxic effect by binding to the tubulins in the neurons and thus be responsible for VIPN.^38^ If vincristine is given as a multi‐day infusion, the tubulin compartment in the blood may not reach its maximum binding capacity, resulting in less overshoot of vincristine and thus less toxicity. In contrast, when given as a 1‐hour infusion, the tubulins in the blood compartment may be saturated regardless, which explains why we did not find less toxicity when vincristine was given as a 1‐hour infusion.

In participants receiving concurrent azoles, one‐hour administration of vincristine resulted in less VIPN compared with push administration: the push group had a twice as high chance of having one point higher on the CTCAE scale. We confirmed this finding from the previously published study.^15^ Azoles are potent CYP3A4 inhibitors.^39^, ^40^ Since vincristine is metabolized by CYP3A4, concurrent azoles and vincristine administration may result in less efficient vincristine elimination. If vincristine is given as a 1‐hour infusion, this may result in a slight protective effect per administration against VIPN, although prolonging vincristine administration most likely mainly affects vincristine distribution and not elimination. Of note, this was an exploratory subgroup analysis and the study was therefore not designed to answer this research question. This may also explain why total CTCAE score was lower in participants in the one‐hour group who received concurrent azoles compared with those not receiving concurrent azoles.

We observed that more participants in the one‐hour group relapsed than in the push group. However, the number of participants with a high risk disease profile was higher in the one‐hour group than in the push group. Furthermore, we do not consider it biologically plausible that 1‐hour infusion of vincristine results in less overshoot of vincristine in the blood and thus lower therapeutic efficacy. All in all, we argue that it is unlikely that the duration of vincristine administration is the cause of the observed difference in treatment outcome between the push and one‐hour group.

The results of this final analysis confirms those of the previously published one‐year analysis.^15^ The protective effect of increasing administration duration in participants receiving concurrent azole treatment persists long‐term; when VIPN has (partially) resolved for most participants. In addition to our previous publication, this analysis also described the treatment outcome of the participants with sufficient follow‐up (at least 3 years). Although these findings should be interpreted with caution, they are relevant for future research on optimizing vincristine administration to reduce toxicity and increase efficacy.

The strength of this study is that we performed longitudinal VIPN assessments using sensitive measurement tools. We assessed the effect of administration duration on VIPN during and after treatment. A weakness of this study is that the study was not adequately powered to detect a difference in treatment outcome, which may hamper the interpretation of the clinical significance of the study. Another weakness of this study is that measurements were performed at different time points after a varying number of vincristine administrations. Since the timing of VIPN assessment in relation to the number and time of vincristine administrations affects VIPN, this likely influenced the results. We corrected for cumulative vincristine dosage and intra‐individual variability, but in this analysis, it was not possible to include the time between VIPN assessment and vincristine administrations. We are planning to investigate this in a follow‐up pharmacokinetic–pharmacodynamic (PK/PD) study. In addition, a median follow‐up of 20 months is relatively short to assess the long‐term effect of randomization on VIPN. However, studies have shown that there is little additional recovery from VIPN as the interval following vincristine administration increases.^4^ Furthermore, patients with nephroblastoma, low‐grade glioma and medulloblastoma received other potential peripheral neurotoxic medication.^21^, ^23^, ^24^ These treatment protocols included carboplatin, whose peripheral neurotoxicity is negligible compared with vincristine, and cisplatin, which may indeed cause peripheral neuropathy but rarely presents with motor or autonomic symptoms.^41^, ^42^, ^43^ Considering the low amount of patients treated with potent neuropathic medication, we argue that neuropathy measured in this study was most likely caused by vincristine. In addition, other drug interactions besides azoles are unlikely. No potent CYP3A4/5 inducers or inhibitors were administered simultaneously with vincristine. Finally, the results were not similar when using the CTCAE and ped‐mTNS, suggesting that these tools measure VIPN symptoms differently. Although we aimed to measure VIPN as reliably as currently possible by using two measurement methods and including severe VIPN, we acknowledge that these are still imperfect representations of the clinical reality of VIPN. The ped‐mTNS is thought to be the best measurement tool for VIPN currently available.^44^, ^45^ Research into VIPN would profit from the development of a tool with good content validity and reliability that can be used in children of all ages.

The VINCA trial assessed the effect of 1‐hour infusion of vincristine in comparison with push injection on the development of VIPN in children with cancer. We found that there was no long‐term effect of administration duration on VIPN. However, in participants receiving concurrent azoles, one‐hour administration was less toxic compared to push administration, suggesting that some children may benefit from one‐hour vincristine administrations. Future studies should aim to clarify the relationship between the PK and PDs of vincristine, including toxicity and efficacy. This will contribute to precision treatment of vincristine in children with cancer.

## AUTHOR CONTRIBUTIONS

**Aniek Uittenboogaard:** Formal analysis (lead); methodology (lead); project administration (equal); software (lead); visualization (lead); writing – original draft (lead); writing – review and editing (lead). **Marleen H. van den Berg:** Conceptualization (equal); funding acquisition (equal); investigation (equal); methodology (equal); project administration (equal); writing – review and editing (equal). **Floor C.H. Abbink:** Investigation (equal); methodology (equal); writing – review and editing (equal). **Jos WR Twisk:** Formal analysis (supporting); methodology (equal); writing – review and editing (equal). **Inge M van der Sluis:** Investigation (equal); writing – review and editing (equal). **Cor van den Bos:** Investigation (equal); writing – review and editing (equal). **Marry M. van den Heuvel‐Eibrink:** Investigation (equal); writing – review and editing (equal). **Heidi Segers:** Investigation (equal); writing – review and editing (equal). **Christophe Chantrain:** Investigation (equal); writing – review and editing (equal). **Jutte van der Werff ten Bosch:** Investigation (equal); writing – review and editing (equal). **Leen Willems:** Investigation (equal); writing – review and editing (equal). **Gertjan J L Kaspers:** Conceptualization (equal); funding acquisition (equal); investigation (equal); methodology (equal); supervision (equal); writing – review and editing (equal). **Mirjam Esther van de Velde:** Investigation (equal); methodology (equal); project administration (equal); supervision (equal); writing – review and editing (equal).

## FUNDING INFORMATION

This research was funded by the Netherlands Organization for Health and Development (pro‐gram Proper Use of Medication; 836021006) and the Belgian Health Care Knowledge Centre (16015).

## CONFLICT OF INTEREST STATEMENT

The authors declare no conflicts of interest.

## ETHICS APPROVAL STATEMENT

Ethical approval was obtained before initiation of this study (Institutional Review Board (IRB) of Amsterdam UMC, location VUmc (IRB number: 2014–268).

## PATIENT CONSENT STATEMENT

Patient consent was obtained from all participants of this study.

## CLINICAL TRIAL REGISTRATION

This clinical trial was registered at EUDRACT (EUDRACT number: 2014–001561‐27).

## Supporting information


Data S1.

## Data Availability

Data is available upon reasonable request.
