# Supplementary material for: Randomized controlled trial on the effect of 1‐hour infusion of vincristine versus push injection on neuropathy in children with cancer (final analysis)
Source: Cancer Med. 2023 Sep 21;12(19):19480–90. doi: 10.1002/cam4.6550 (PMC10587928; doi:10.1002/cam4.6550)
Supplement: Supplementary file 1 — Data S1. [file CAM4-12-19480-s001.docx]

**Supplementary tables**

**Table S1.** Characteristics of participants in the randomization groups (push- and one-hour administration of vincristine) per age at start of the study.

|  | **Push administration group (n = 45)** | | **One-hour administration group (n = 45)** | |
| --- | --- | --- | --- | --- |
|  | **2-4 years old (n = 11)** | **5-18 years old (n = 34)** | **2-5 years old (n = 12)** | **5-18 years old (n = 33)** |
| Male, n (%) | 4 (36.4) | 20 (58.8) | 6 (50) | 20 (60.1) |
| Disease, n (%)  ALL  Hodgkin lymphoma  Medulloblastoma  Low-grade glioma  Wilms tumor  Rhabdomyosarcoma | 7 (63.6)  1 (9.1)  0 (0)  2 (18.2)  1 (9.1)  0 (0) | 22 (64.7)  10 (29.4)  1 (2.9)  0 (0)  1 (2.9)  0 (0) | 7 (58.3)  0 (0)  0 (0)  0 (0)  5 (41.7)  0 (0) | 22 (66.7)  7 (21.2)  1 (3.0)  0 (0)  1 (3.0)  2 (6.1) |
| Racial background, n (%)  White  Non-white | 10 (90.1)  1 (9.1) | 27 (79.4)  7 (20.6) | 10  2 | 26 (78.8)  7 (21.2) |
| Follow-up duration in months (mean (SD)) | 23.1 (9.0) | 20.4 (9.9) | 20.1 (10.1) | 17.6 (10.2) |
| Vincristine dose reduction or omission, n (%) | 0 (0) | 0 (0) | 2 (4.4) | 0 (0) |

**Table S2.** Adjusted analysis on the effect of one-hour administration in comparison with push administration of vincristine on the development of VIPN over time, including age, sex, BSA normalized cumulative vincristine dosage, diagnosis, and racial background.

|  | **Total group (n = 89)** | | **Subgroup of participants without concurrent azole treatment (n = 75)** | | **Subgroup of participants with concurrent azole treatment (n = 14)** | |
| --- | --- | --- | --- | --- | --- | --- |
| **Continuous outcomes** | **Rate ratio (95% CI)** | **p-value** | **Rate ratio (95% CI)** | **p-value** | **Rate ratio (95% CI)** | **p-value** |
| Total CTCAE | 0.92 (0.66 to 1.27) | 0.61 | 1.02 (0.70 to 1.48) | 0.91 | 0.40 (0.24 to 0.66) | 0.0003 |
| Total ped-mTNS* | 0.96 (0.61 to 1.52) | 0.87 | 1.01 (0.58 to 1.75) | 0.97 | 1.57 (0.74 to 3.35) | 0.24 |
| **Dichotomized outcomes** | **OR (95% CI)** | **p-value** | **OR (95% CI)** | **p-value** | **OR (95% CI)** | **p-value** |
| CTCAE | 0.73 (0.41 to 1.32) | 0.30 | 0.87 (0.46 to 1.65) | 0.67 | 0.02 (0.001 to 0.59) | 0.02 |
| Ped-mTNS* | 0.99 (0.50 to 1.96) | 0.99 | 0.96 (0.45 to 2.05) | 0.93 | 1.38 (0.37 to 5.18) | 0.63 |
| Severe VIPN according to CTCAE | 0.68 (0.22 to 2.03) | 0.48 | 0.93 (0.16 to 5.31) | 0.93 | 0.17 (0.02 to 1.27) | 0.08 |

Reference group is push administration. CTCAE: 350 observations in 89 participants and ped-mTNS: 221 observations in 60 participants. One participant could not be included in these analyses since only a baseline measurement was available. * Total group consisted of 60 participants (without concurrent azole treatment: 49, with concurrent azole treatment: 11).


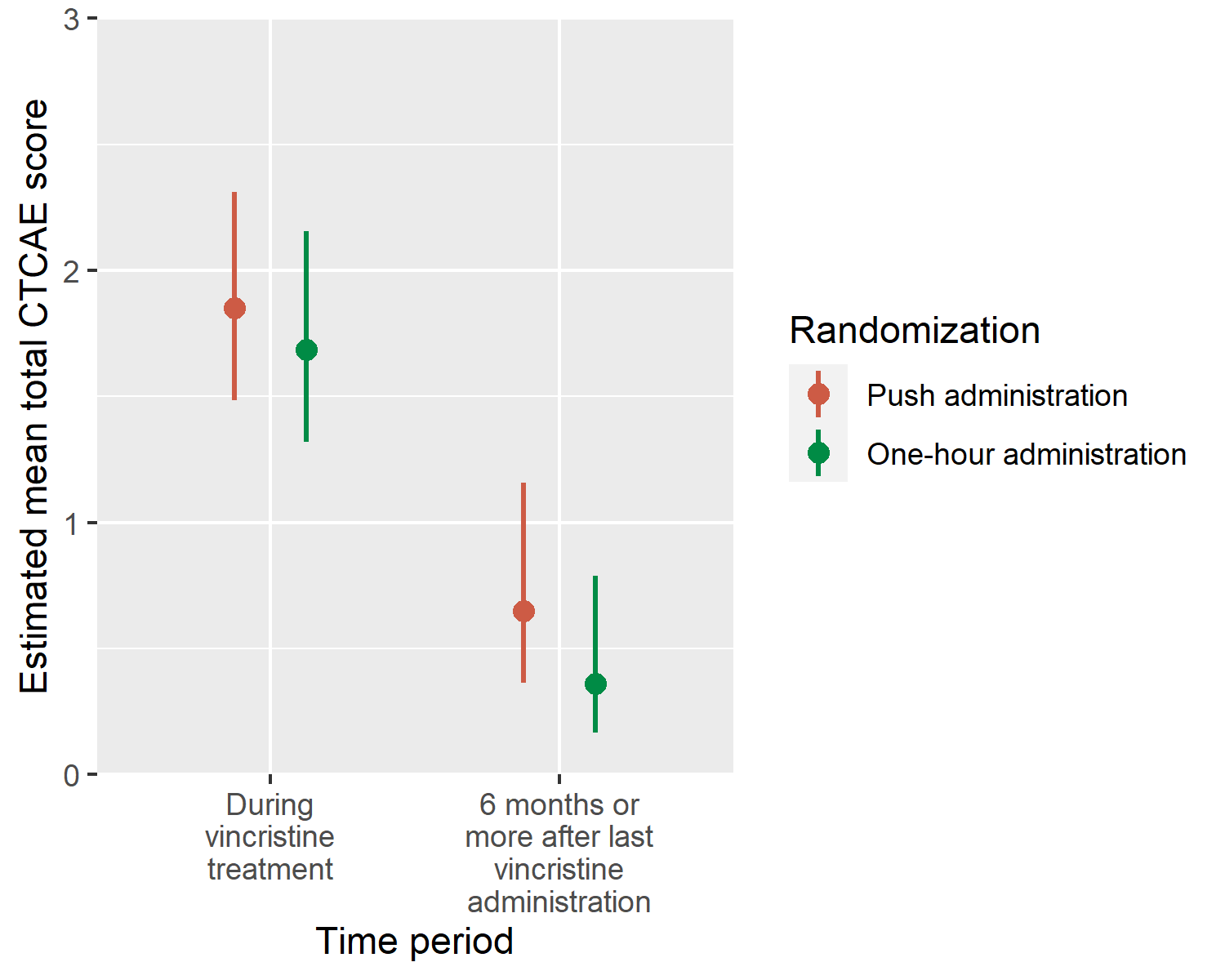
**Supplemental Figure S1.** Difference in VIPN according to the CTCAE between the push and one-hour group during and ≥ six months after last vincristine administration (rate ratio during vincristine treatment 0.91, 95% CI 0.73 to 1.13, p=0.55, rate ratio ≥ six months after last vincristine administration 0.56, 95% CI 0.24 to 1.28, p=0.17).

**Table S3.** Risk stratification and treatment protocol of participants included in the randomization groups.

|  | **Push (n = 45)** | **One-hour (n = 45)** |
| --- | --- | --- |
| ALL (ALL-11 or EsPHALL protocol)  SR  MR  HR  Ph-ALL | 4 (8.9)  7 (15.6)  1 (2.2)  1 (2.2) | 2 (4.4)  8 (17.8)  1 (2.2)  2 (4.4) |
| ALL (EORTC-CLG 58081 protocol)  VLR  AR-2T  AR1  AR2-B  VHR | 2 (4.4)  3 (6.7)  8 (17.8)  0 (0)  3 (6.7) | 1 (2.2)  2 (4.4)  9 (20)  1 (2.2)  3 (6.7) |
| Hodgkin lymphoma (EuroNet-PHL-C1 or C2 protocol)  TL1  TL2  TL3 | 4 (8.9)  5 (11.1)  2 (4.4) | 3 (6.7)  3 (6.7)  1 (2.2) |
| Wilms tumor (SIOP Wilms 2001 protocol)  LR  HR  Other * | 1 (2.2)  1 (2.2)  0 (0) | 2 (4.4)  3 (6.7)  1 (2.2) |
| Low-grade glioma (SIOP-LGG 2004 protocol) | 2 (4.4) | 0 (0) |
| RMS HR (EpSSG RMS 2005 protocol) | 0 (0) | 2 (4.4) |
| Medulloblastoma (ACNS0331 or 0332 protocol)  SR  HR | 1 (2.2)  0 (0) | 0 (0)  1 (2.2) |

* After initial diagnosis with a Wilms tumor, the participant received a different diagnosis during treatment (malignant rhaboid tumor of the kidney with pulmonary and lymph node metastases) and was subsequently excluded from the study. All vincristine administrations were given as one-hour infusions.
